# To Medical Men

**Published:** 1856-06

**Authors:** 


We would again appeal to our friends, to give us the results of their observation and experience for the columns of the Journal. We are happy in the receipt of a well written article, and as our brethren are a *reading* class of men, they should also be found a *writing* profession. Let no one think that these remarks are meant for another then in his mind's eye, but let him be assured they are aimed, pointedly and particularly, at himself. Let him not shrug off the responsibility upon other shoulders, but go to work with earnestness, and very soon our "drawer" will be full to repletion.

The leisure afforded by the prevalent *alarming* state of the health was propitious for collecting and collating the facts of cases which have heretofore passed under the notice of each, and of which notes have been made and kept, but because of pressing and continued duty and labor, time was not afforded in which to write them out.

Our friends and patrons will not force us to appeal to them again.

